# An Overlooked Formulation Variable: Solvent Reshapes DNA Delivery

**DOI:** 10.1021/acsmacrolett.6c00160

**Published:** 2026-05-20

**Authors:** Yinghao Li, Bei Qiu, Liang Yao, Jiahao Liu, Zishan Li, Xinhui Wang, Jing Lyu, Wenxin Wang

**Affiliations:** † Charles Institute of Dermatology, School of Medicine, 8797 University College Dublin, D04 V1W8 Dublin, Ireland; ‡ Yusuf Hamied Department of Chemistry, University of Cambridge, Cambridge CB21EW, United Kingdom; § Centre for Regenerative Medicine, Institute for Regeneration and Repair, the University of Edinburgh, Edinburgh EH16 4UU, United Kingdom; ∥ Department of Chemistry, University of Manchester, Oxford Road, Manchester M13 9PL, United Kingdom; ⊥ School of Mechanical Engineering, State Key Laboratory of Traction Power, 56711 Southwest Jiaotong University, Chengdu 610031, China; ○ Institute of Precision Medicine (AUST-IPM), 91594 Anhui University of Science and Technology, Huainan 232001, China

## Abstract

Nonviral polymeric vectors offer a tunable platform for nucleic acid delivery, yet formulation variables beyond polymer structure remain underexplored. Here, we systematically investigate the role of stock solvent in shaping gene delivery performance using end-capped highly branched poly­(amino ester) (HPAE) vectors. By combining a structured experimental design with an interpretable machine learning framework, we reveal that transfection efficiency is primarily governed by polymer features, while solvent properties modulate performance through strong polymer–solvent interactions. Descriptor-level analysis identifies key molecular features associated with high-performing formulations, highlighting the importance of polymer size, charge density, and hydrophobic–polar balance. Selected polymer–solvent combinations enable efficient CFTR protein restoration in cystic fibrosis disease cells and achieve robust *in vivo* transgene expression following systemic administration in mice. Overall, this work establishes stock solvent as a critical and tunable formulation parameter and provides a data-driven framework for optimizing polymeric gene delivery systems.

## Graphical abstract

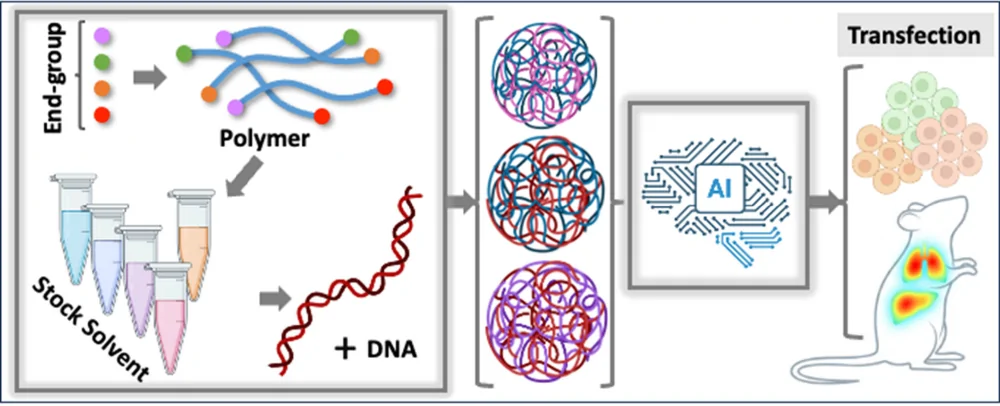

Nucleic acid therapeutics provide a powerful avenue for treating diseases that remain difficult to address with conventional small molecules and biologics.^1–4^ However, their clinical translation is severely constrained by delivery barriers: nucleic acids are large, negatively charged, and prone to degradation, and must overcome extracellular clearance, cellular uptake limitations, and intracellular trafficking obstacles to reach the appropriate subcellular compartment for functional expression.^5^ Although viral vectors can achieve high delivery efficiency, concerns regarding immunogenicity, manufacturing complexity, packaging limitations, and repeat dosing continue to drive the development of nonviral delivery systems.^6,7^ Among these, cationic polymers have attracted considerable attention due to their synthetic tunability, scalable production, and ability to form electrostatic complexes with DNA, thereby facilitating cellular internalization and endosomal escape.^8–10^

Poly­(β-amino ester)­s (PAEs) and related PAE platforms represent a widely used class of polymers for gene delivery owing to their biodegradability and modular synthesis, as well as their capacity to tune charge density, hydrophobicity, and buffering ability through rational monomer selection and terminal group modification.^11–14^ A substantial body of evidence has demonstrated that relatively subtle structural changes—particularly in end-group chemistry—can markedly influence DNA binding, complex stability, cytotoxicity, and transfection outcomes.^15–17^ This pronounced structure–property sensitivity presents both opportunities and challenges: while combinatorial polymer libraries can yield highly effective candidates, establishing robust design principles remains complex due to the multiparameter dependence on polymer structure, formulation conditions, and biological context.

Importantly, polymer-mediated gene delivery is not dictated solely by polymer structure and polymer-to-DNA ratio. Practical formulation variables—often regarded as secondary—can substantially modulate the assembly pathway and final physicochemical properties of polymer/DNA complexes. One such variable is the stock solvent used to prepare polymer solutions prior to complex formation. In a typical workflow, polymers are dissolved in an organic solvent (or solvent mixture) to generate a concentrated stock solution, which is subsequently diluted into aqueous media during formulation.^18–20^ Differences in stock solvent polarity, hydrogen-bonding properties, and hydrophobicity can affect polymer solvation, preaggregation state, and the kinetics of complex formation upon mixing with DNA. These upstream effects may propagate to changes in particle size distribution, surface charge, and colloidal stability, ultimately influencing cellular interactions and transfection efficiency. Despite its potential impact, the role of stock solvent has received far less systematic attention compared with polymer chemistry or polymer/DNA ratio, and clear guidelines for selecting or optimizing stock solvents remain limited.

To fill this gap, we tested a set of combinations by pairing different end-capped highly branched PAE (HPAE) vectors with different stock solvents, and we evaluated transfection performance across several polymer-to-DNA weight ratios. Because polymer structure, solvent properties, and formulation ratio may exhibit nonlinear and interaction-dependent effects, conventional one-factor-at-a-time analysis is insufficient to reveal the governing patterns. Therefore, we employed an interpretable machine learning framework integrating physicochemical descriptors, chemical fingerprints of both polymers and solvents, and formulation ratio to predict and rationalize transfection outcomes. This approach quantifies the relative contributions of polymer and solvent features, identifies key descriptors associated with performance, and visualizes polymer–solvent interaction patterns. Beyond reporter gene expression, we further validated translational potential in a cystic fibrosis (CF) disease model, a monogenic inherited disorder caused by loss-of-function mutations in CFTR,^21,22^ and evaluated *in vivo* expression kinetics and organ biodistribution in immunocompetent mice following tail-vein injection of luciferase plasmid complexes. Overall, this study identifies stock solvent as a key and tunable formulation variable, elucidates the polymer–solvent–ratio determinants of transfection in a data-driven manner, and demonstrates the functional and *in vivo* relevance of optimized polymer–solvent combinations.

An HPAE–stock solvent formulation library was first constructed to evaluate how polymer end-group chemistry, stock solvent identity, and polymer:DNA ratio influence *in vitro* gene delivery. Using the classic monomer combination of 1,4-butanediol diacrylate (BDA), 5-amino-1-pentanol (AP), and pentaerythritol tetraacrylate (PTTA), an HPAE backbone was synthesized. The resulting polymer was then divided into eight equal portions and end-capped with a series of amines commonly used in polymeric vectors (Figure 1), yielding HPAE-A to HPAE-H. Except for slight degradation observed in HPAE-F, the final molecular weights of HPAE-A to HPAE-H were comparable (weight-average molecular weight (*M*_w_) around 10 to 15 kDa, Figure S1, Table S1). ^1^H NMR spectra (Figure S2) confirmed successful end-capping with no residual vinyl groups detected. To investigate the effects of different stock solvents, the obtained HPAEs were dissolved at 100 mg/mL in different solvents (Figure 1): (1) dimethyl sulfoxide, (2) *N*-methyl-2-pyrrolidone, (3) acetone, (4) 1,3-butanediol, (5) ethyl acetate, (6) butanone, (7) methyl isobutyl ketone, and (8) ethanol.

**Figure 1:**
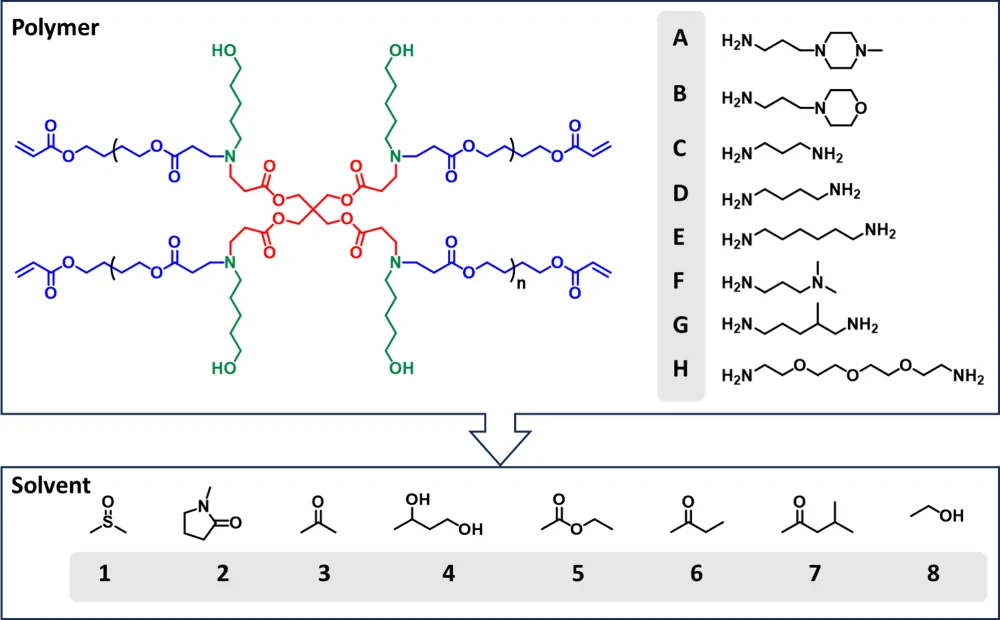
Molecular structures of the polymers and end-capping groups, as well as the molecular structures of the stock solvents.

To explore the gene delivery behaviors of different polymers stored in different stock solvents, CFBE disease cells were used as the transfection model, and a commercial plasmid gWiz encoding GFP was delivered. Considering that polymers with different structures may have different optimal dosages, four polymer:DNA weight ratios (w/w) were tested: 10:1 (Figure 2A), 20:1 (Figure 2B), 30:1 (Figure 2C), and 40:1 (Figure 2D). To further examine the role of stock solvent, HPAE-A was selected as a representative system to evaluate solvent-dependent effects on polyplex physicochemical properties. The hydrodynamic size (Figure S3), zeta potential (Figure S4), and colloidal stability over time (Figure S3) were characterized. However, no clear trends were observed, and the variations in these properties did not directly correlate with the transfection outcomes. Notably, polyplexes prepared in solvent (3) acetone and solvent (7) methyl isobutyl ketone exhibited relatively poor stability. Overall, the optimal dosage for most polymer/stock solvent combinations was 20:1 or 30:1. However, no specific solvent or polymer structure showed a clear advantage, and there was no obvious linear structure–transfection performance relationship. In addition, the direct effects of residual solvents on cells were evaluated (Figure S5) by introducing additional solvents into the culture medium after transfection (HPAE-A) at a solvent:DNA ratio of 400:1. While negligible effects on cell viability were observed, transfection efficiency was differentially affected depending on the solvent type. These results suggest that organic solvents may influence transfection not only through modulation of polyplex properties but also via effects on cellular behavior, leading to complex and coupled outcomes. In addition, the chemical structures of both the polymers and the solvent molecules varied substantially, making direct structure–transfection analysis quite challenging and suggesting that more powerful analytical approaches are needed to interpret the results. On the other hand, most systems exhibited good biocompatibility. However, a few systems—especially those stored in solvent 7—showed significant toxicity at high polymer:DNA ratios (Figure S6).

**Figure 2:**
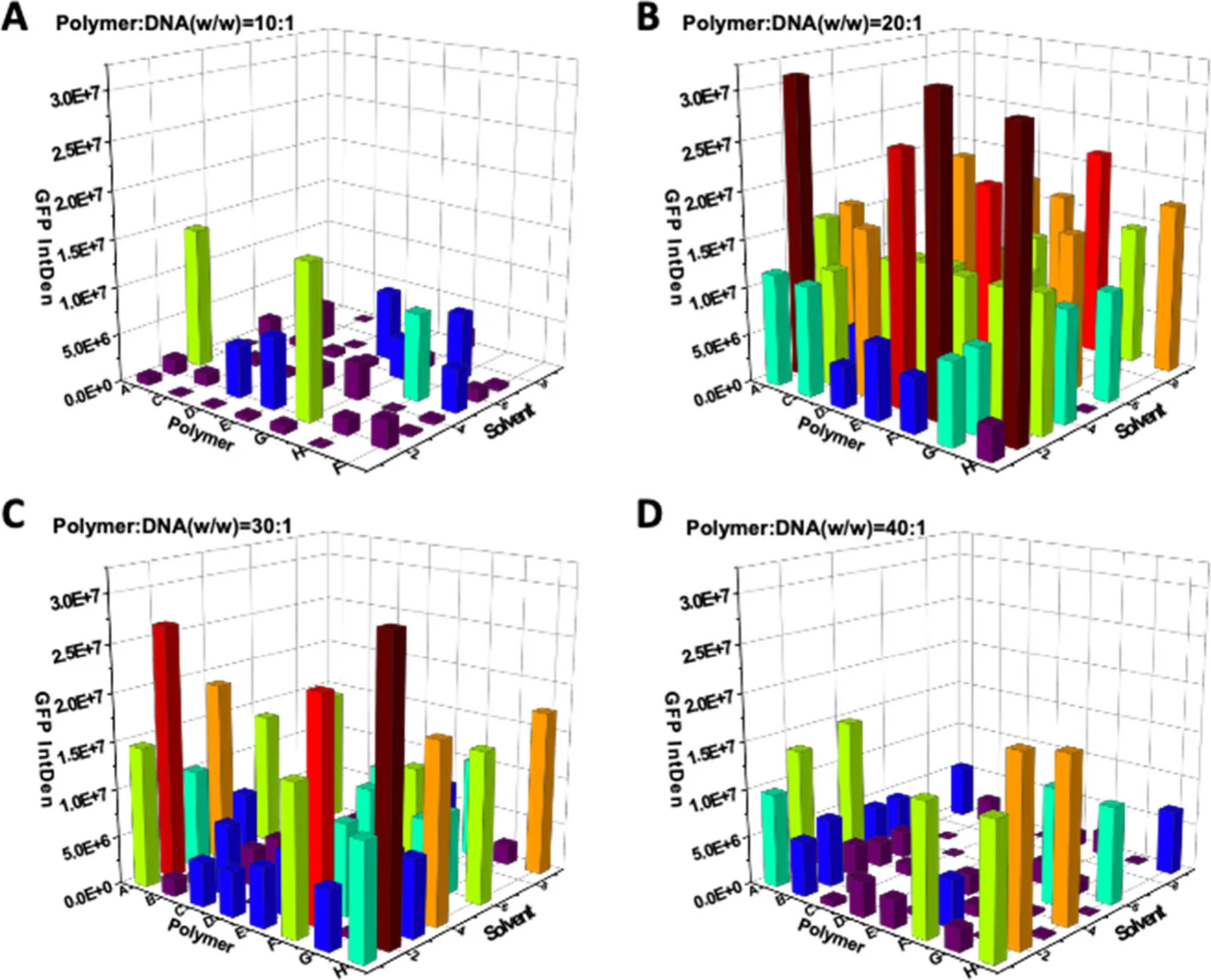
*In vitro* transfection performance evaluation of different polymer/solvent system with polymer: DNA weight ratio (w/w) as (A) 10:1, (B) 20:1, (C) 30:1, and (D) 40:1.

To obtain effective conclusions and uncover the underlying polymer/solvent molecular structure–transfection performance relationships behind the experiments, a supervised machine learning workflow was established to model and interpret the effects of polymer chemical properties, stock solvent type, and formulation ratio on *in vitro* transfection performance.^23^ Considering that all polymers shared the same backbone, information from the end-capping amines (Figure 1) was selected as the structural representation of HPAE-A to -H, and the molecular structures of the stock solvents were re-encoded in the form of SMILES strings. A combined descriptor–fingerprint strategy was then applied to generate molecular representations. For both polymers and solvents, physicochemical descriptors were recalculated, including molecular weight (MolWt), hydrophobicity (LogP), topological polar surface area (TPSA), hydrogen bond donor/acceptor counts (HBD/HBA), rotatable bonds (RotBonds), ring count (RingCount), and heavy atom count (HeavyAtom). In addition, to characterize cationic-related properties of the materials, the number of nitrogen atoms (N_count), formal charge (FormalCharge), and nitrogen density normalized by molecular weight (N_per_MW) were further extracted. For structural representation, Morgan circular fingerprints were encoded separately for polymers and solvents. Model training was performed using a random forest regressor, and predictive performance was evaluated via 5-fold cross-validation. After cross-validation, the model was refitted using the full data set for subsequent analysis.

First, we investigated how “polymer structure–stock solvent–formulation ratio” jointly determines transfection efficiency. Grouped feature importance (Figure 3A) showed that model predictions were mainly driven by polymer-related features, among which the polymer:DNA ratio was the most influential factor, followed by solvent molecular fingerprints and polymer molecular descriptors. In contrast, solvent descriptors and polymer structural fingerprints contributed less, suggesting that transfection performance depends not only on the polymer itself but is also strongly regulated by the formulation window. Furthermore, the top 15 descriptors (Figure 3B) revealed the key physicochemical properties influencing transfection efficiency: the most important features were concentrated in polymer size and composition (polymer_HeavyAtom, polymer_MolWt) and solvent molecular weight (solvent_MolWt). Meanwhile, polymer polarity-related parameters (polymer_TPSA), polymer hydrophobicity (polymer_LogP), and solvent hydrophobicity (solvent_LogP) also ranked highly, indicating that the size of the polymer end groups and the hydrophobic–polar balance are core determinants of carrier transfection performance. Finally, the polymer–solvent interaction heatmap (Figure 3C) further confirmed that the system exhibited strong combination-dependent interactions: the average transfection efficiency of different polymers showed clear stratification under different solvent conditions, demonstrating that high transfection performance cannot be fully explained by a single polymer or a single solvent, but is instead shaped by their matching relationship. This provides direct evidence supporting subsequent screening and optimization of high-performance systems through polymer–solvent synergistic tuning.

**Figure 3:**
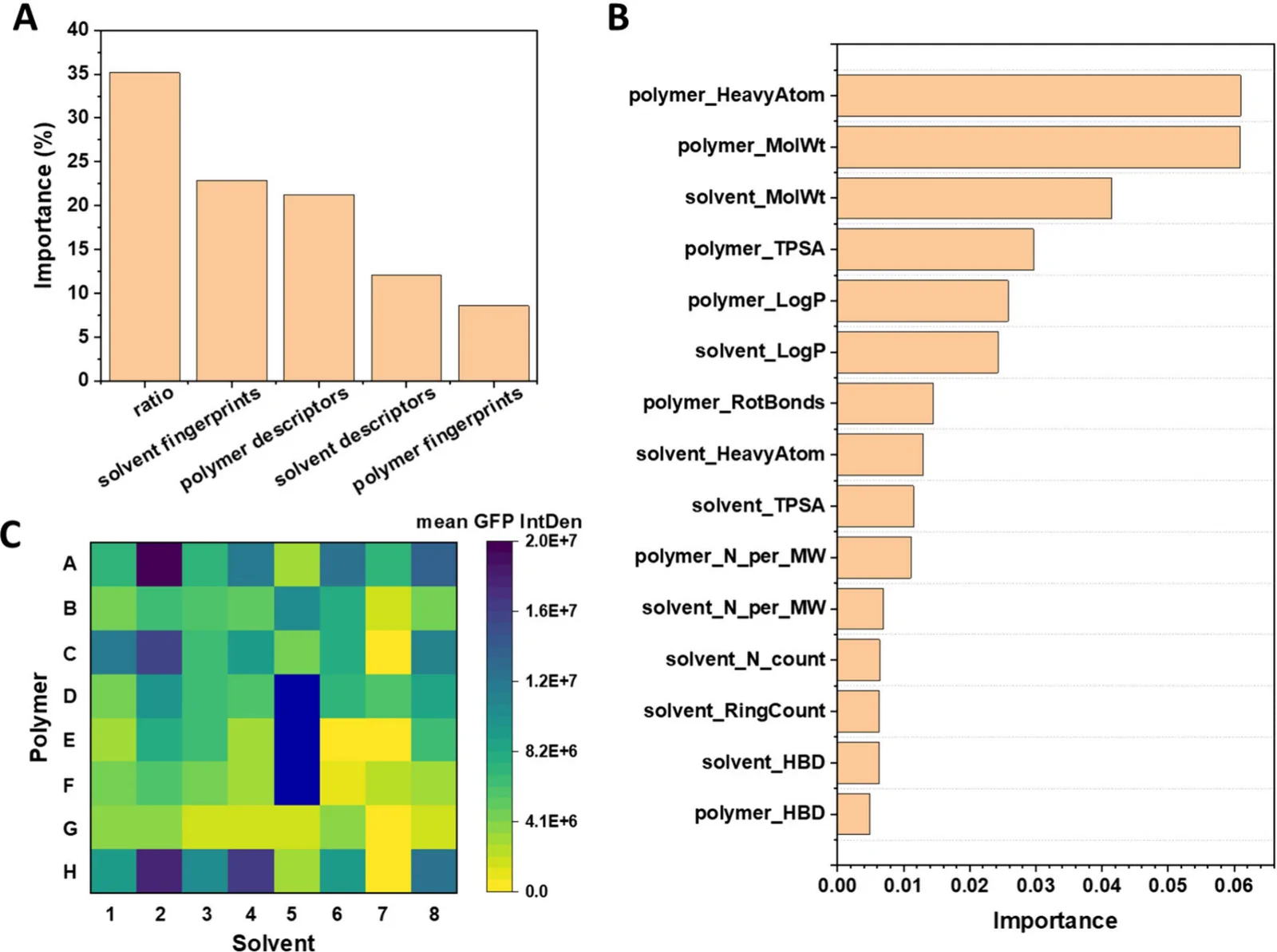
Feature importance analysis of polymer and solvent properties. (A) Grouped feature importance reveals the contributions from polymer, solvent, and ratio descriptors/fingerprints. (B) Top 15 molecular descriptors ranked by Random Forest feature importance. (C) Polymer–solvent combination interaction heatmap (average transfection efficiency for each combination). Dark blue in the center indicates missing experimental data for the corresponding combinations.

As demonstrated in Figure 3, transfection efficiency is not determined by a single factor, but rather results from the synergistic effects of multiple variables, dominated by the formulation ratio, together with polymer physicochemical properties and solvent structure–mediated regulation. Next, Figures 4 and S7 further elaborate on the key variables identified by the model in order to illustrate their relationships with transfection efficiency.

**Figure 4:**
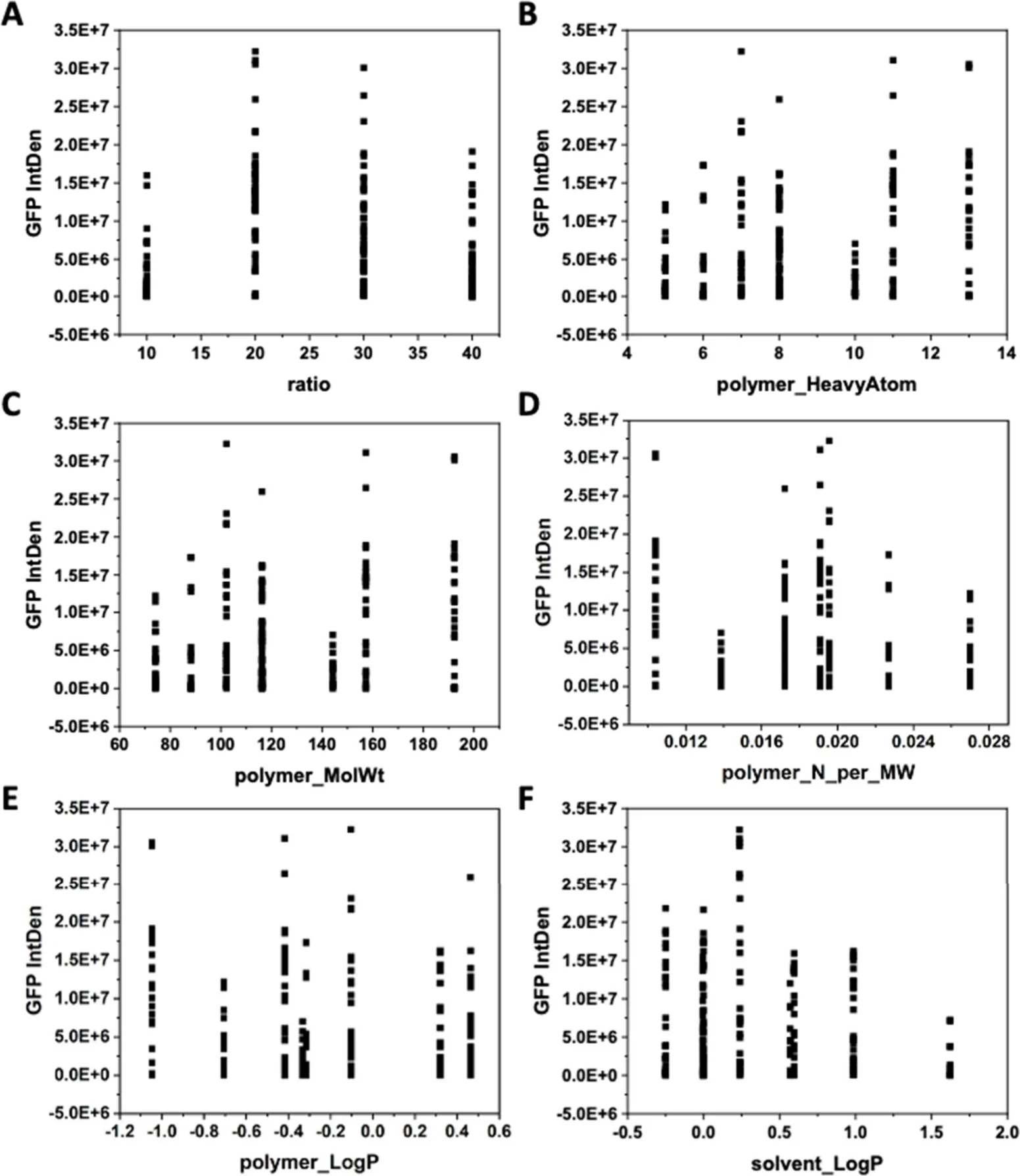
Relationships between polymer/solvent features and transfection efficiency. (A) Polymer:DNA ratio versus transfection efficiency. (B) Number of heavy atoms in the polymer end groups (HeavyAtom), (C) molecular weight of the end groups (MolWt), (D) polymer nitrogen density (N_per_MW), (E) polymer hydrophobicity (LogP), and (F) stock solvent hydrophobicity (LogP) versus transfection efficiency.

Figure 4A first shows that the polymer:DNA weight ratio has a clear impact on transfection efficiency, indicating that the formulation window is a critical external condition determining system performance. Different polymers may exhibit drastically different efficiency levels at different w/w ratios, suggesting that high-performing formulations require a balance among charge neutralization, complex stability, and cytotoxicity, rather than a simple linear improvement with increasing polymer dosage.^24,25^ Notably, high-performing formulations were mainly concentrated at ratios of 20:1 and 30:1.

Next, Figure 4B–D further explain this phenomenon from the perspective of polymer structure. The trends between the number of heavy atoms in the polymer end groups (HeavyAtom, Figure 4B) and the molecular weight of the end groups (MolWt, Figure 4C) indicate that high transfection performance consistently requires end groups with sufficient length and size.^26–28^ As the end groups become larger, the number of experimental groups achieving high transfection performance also increases. Meanwhile, the relationship between polymer nitrogen density (N_per_MW, Figure 4D) and transfection efficiency highlights the critical role of cationic density during transfection: a moderate positive charge is beneficial, whereas excessively high positive charge leads to a marked reduction in high-performance formulations, suggesting the existence of a potential optimal range.^29,30^

Finally, Figure 4E,F extend this trend to hydrophobicity. Polymer LogP and stock solvent LogP together suggest that the material system must maintain a reasonable hydrophobic–hydrophilic balance. Overall, high-performing polymers tend to be relatively hydrophilic (LogP < 0), while effective stock solvents tend to exhibit mild hydrophobicity (0 < LogP < 0.5). However, transfection performance decreases significantly as solvent hydrophobicity increases. This observation further supports the combination dependence revealed in Figure 3C: the solvent is not only a medium for dissolving the carrier, but may also influence polymer solvation, complex assembly pathways, or the resulting nanostructures, thereby ultimately altering transfection performance.

Based on the results above, it can be confirmed that improving transfection efficiency cannot be achieved by a single factor alone (e.g., only increasing cationic density or only adjusting hydrophobicity). Instead, it is more likely driven by an “optimal synergistic window” arising from the combined effects of polymer structural features, solvent chemical properties, and formulation ratio. Building on this, Figure 5 further places these factors into a broader chemical space perspective and aims to address what systematic molecular-property differences exist between high- and low-performing systems, thereby extracting more generalizable design rules.

**Figure 5:**
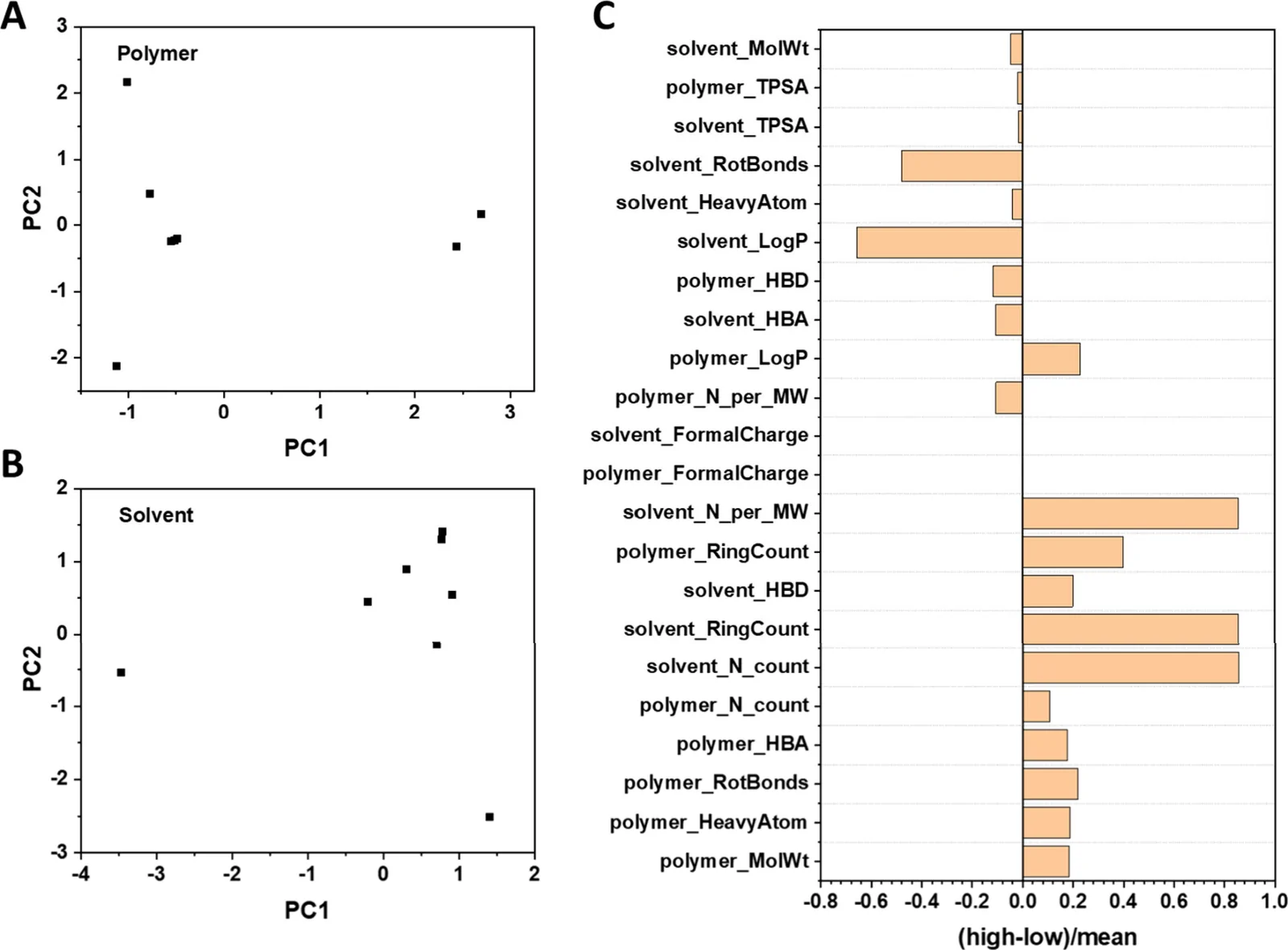
Chemical space projection and categorical analysis of polymers and stock solvents. Fingerprint-based chemical space projection (PCA) of (A) polymers and (B) stock solvents. (C) Descriptor differences between high and low transfection performers, normalized as (high – low)/mean.

First, Figure 5A,B show PCA projections based on the Morgan fingerprints of the polymer and solvent molecules, respectively. Their dispersed distributions indicate that the investigated polymer library possesses a certain degree of structural diversity across chemical space. Next, based on the top 20% and bottom 20% of transfection performance, the systems were divided into high performers and low performers (Figure S8). On this basis, Figure 5C compares molecular descriptor differences between high and low performers (normalized as (high–low)/mean), translating “chemical space differences” into interpretable property trends. The results show that high-transfection systems exhibit clear enhancement trends in polymer structural characteristics compared with low-performing systems, including larger end-group scale and structural complexity (polymer_MolWt and polymer_HeavyAtomCount increased by ∼18–19%), higher chain flexibility/conformational freedom (polymer_RotBonds increased by ∼22%), and a higher proportion of ring structures (polymer_RingCount increased by ∼40%). Meanwhile, high-performing polymers also show a stronger tendency toward hydrophobicity (polymer_LogP increased by ∼23%), suggesting that moderate hydrophobic segments may help enhance membrane interactions or facilitate intracellular delivery processes.^27,31–33^ Notably, although polymer_N_count slightly increased (∼11%), polymer_N_per_MW decreased in the high-performing group (around – 10%), indicating that high efficiency does not simply rely on higher nitrogen density per unit mass.

In contrast, the solvent-related differences exhibit a more directional signal. Solvents associated with high-transfection systems show significantly lower hydrophobicity (solvent_LogP markedly decreased), together with slightly lower RotBonds, MolWt, and HeavyAtomCount overall, suggesting that high-performing formulations tend to favor relatively smaller, more rigid, and more hydrophilic/polar stock solvent environments. In addition, solvent_N_count and solvent_N_per_MW are significantly higher in the high-performing group, indicating that nitrogen-containing functional groups in the solvent may correlate with improved formulation performance in this data set, potentially reflecting the importance of hydrogen bonding or polar interactions in maintaining system stability and delivery efficiency.^34–36^

The above studies not only identified key structural and formulation factors that influence transfection efficiency, but also further screened several representative, high-potential delivery systems for validation in disease-relevant models. To evaluate whether these systems could support therapeutic gene delivery and enable functional protein expression, multiple polymer–stock solvent combinations were selected to deliver a therapeutic CFTR plasmid in the CFBE disease cell model, with CFTR protein restoration used as the end point. Figure 6A shows the Western blot results of CFTR after treatment with different delivery systems. Compared with the control, some delivery systems significantly enhanced the CFTR protein expression, far exceeding that of positive control- human bronchial epithelial (HBE) cells, indicating that these systems not only enable nucleic acid entry into cells but also promote subsequent expression processes and generate therapeutically protein. Furthermore, the semiquantitative analysis in Figure 6B demonstrates that the selected optimal polymer–solvent combinations induced higher levels of CFTR expression in CFBE cells, reaching up to 22-fold that of HBE cells. These results indicate that the identified polymer–solvent synergy is not limited to reporter readouts, but can translate into restoration of a disease-relevant transgene product.

**Figure 6:**
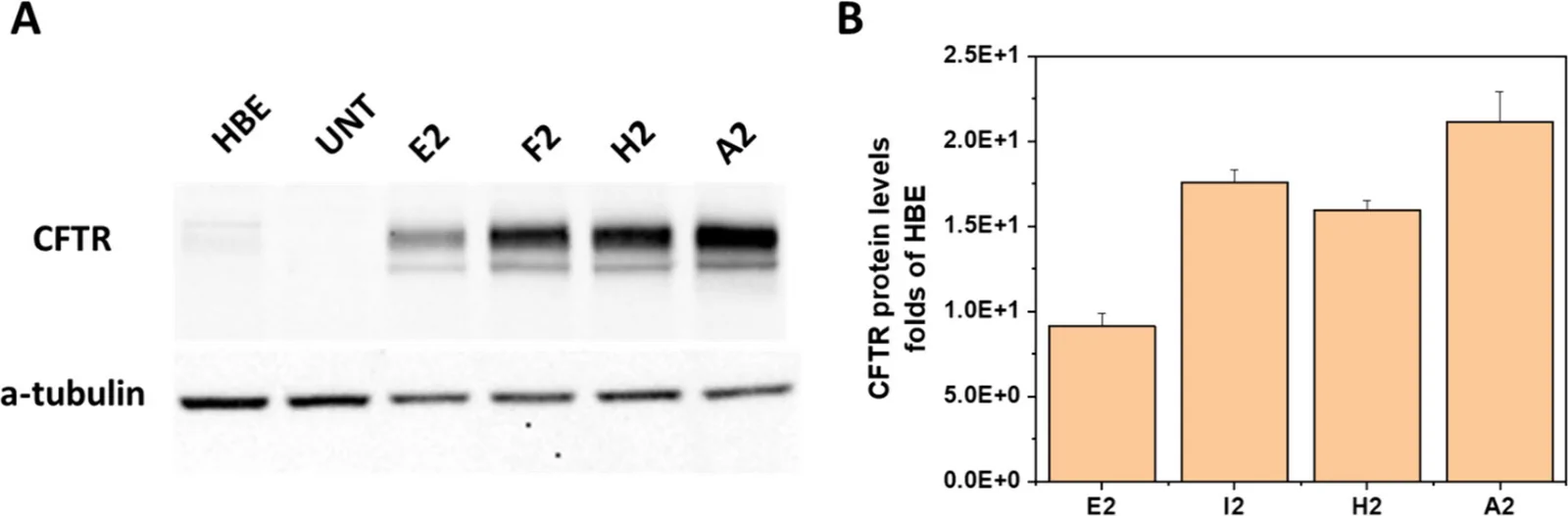
CFTR protein restoration after transfection using different HPAE polymer–solvent formulations, where letters indicate the polymer identity and numbers indicate the solvent identity (e.g., E2 means HAPE-E in solvent 2). (A) Western blot analysis of CFTR expression. (B) Semiquantitative densitometric analysis of CFTR levels.

The transfection capability, expression kinetics, and organ distribution profiles of the optimized delivery systems were further evaluated under the complex *in vivo* environment. To this end, plasmids encoding luciferase were complexed with the selected polymer-based delivery systems and administered to immunocompetent mice via tail-vein injection, followed by longitudinal tracking of *in vivo* expression signals at different time points. Figure 7A shows whole-body bioluminescence imaging results at 6, 24, and 48 h postinjection, together with bioluminescence distributions in major organs. Overall, Figure 7 demonstrates that different polymer–stock solvent delivery systems induced distinct time-dependent luciferase expression kinetics *in vivo*. The *in vivo* bioluminescence imaging results indicate that the luminescence signal could be detected as early as 6 h after administration, reached a more pronounced expression level at 24 h, and then markedly declined by 48 h. This suggests a dynamic expression profile characterized by rapid onset, a midterm peak, and a late-stage decrease following plasmid delivery *in vivo*.^37,38^ Consistently, organ imaging further revealed a clear preference for luciferase expression in the lungs and liver. As a reference control, the A1­(HPAE-A in solvent 1, DMSO) formulation was evaluated in Figure S9, showing that luciferase expression peaked at 6 h postinjection and was predominantly localized in the spleen.

**Figure 7:**
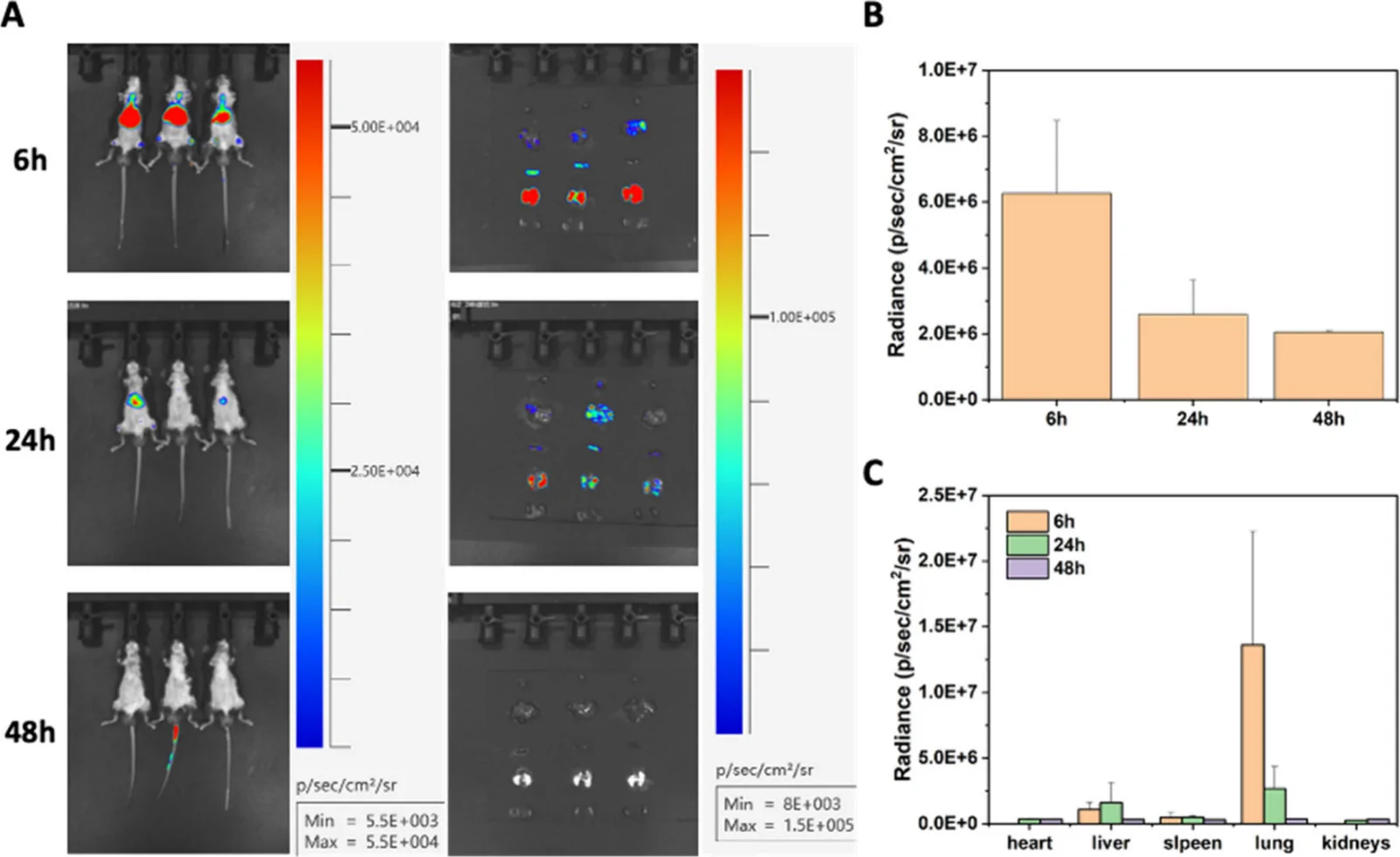
*In vivo* transfection performance evaluation with A2 (HPAE-A in solvent 2). (A) Bioluminescence images of immunocompetent mice and luminescence images of major organs at different time points after tail-vein injection of polymer complexes. Organs from top to bottom are heart, liver, spleen, lung, and kidney. (B, C) Quantitative analysis of the corresponding bioluminescence signals from (A).

Further quantitative analyses (Figure 7B,C) statistically confirmed these trends: the optimized delivery systems generated the highest luminescence signals at 24 h, while the signal decrease at 48 h indicated that sustained expression was difficult to maintain over time. Overall, these *in vivo* results validate that the data-driven selected polymer–stock solvent systems can achieve effective systemic transfection and expression in immunocompetent mice, exhibiting quantifiable time dependence and organ distribution characteristics. This provides key evidence supporting their *in vivo* feasibility for further therapeutic gene delivery applications. While *N*-methyl-2-pyrrolidone (NMP, solvent 2) showed strong performance as a stock solvent, its known safety concerns warrant consideration, and more biocompatible alternatives will be explored in future work.

In this work, we systematically investigated how polymer chemistry, stock solvent identity, and formulation ratio jointly determine nucleic acid delivery outcomes using end-capped HPAE vectors as a model platform. By coupling high-throughput *in vitro* transfection screening with an interpretable machine learning workflow, we revealed that polymer structural features are the primary drivers of transfection performance, while solvent properties provide important modulation through interaction-dependent effects. Feature importance and high/low performer analysis enabled extraction of key molecular descriptors associated with improved transfection, supporting the concept that optimal delivery requires coordinated tuning of polymer size/architecture, charge-related features, and hydrophobic–polar balance rather than maximizing any single parameter. Beyond *in vitro* reporter readouts, selected polymer–solvent formulations achieved therapeutic relevance by regenerating CFTR protein expression in cystic fibrosis disease cells, and further demonstrated measurable systemic transgene expression *in vivo* following intravenous administration. Overall, our findings highlight stock solvent as an overlooked but actionable variable in polymeric gene delivery formulation and provide a data-driven framework to accelerate rational selection and optimization of polymer–solvent combinations for translational nucleic acid therapeutics.

## Supplementary Material



## Notes

Supplementary figures show GPC traces, NMR spectra, cell viability measurement results, size and zeta potential analysis results, figures of relationships between polymer/solvent features and transfection efficiency, *in vivo* transfection images (PDF)
