# Supplementary material for: An Overlooked Formulation Variable: Solvent Reshapes DNA Delivery
Source: ACS Macro Lett. 2026 May 20;15(9):1199–207. doi: 10.1021/acsmacrolett.6c00160 (PMC13585124; doi:10.1021/acsmacrolett.6c00160)
Supplement: Supplementary file 1 [file mz6c00160_si_001.pdf]

SUPPORTING INFORMATION

An overlooked formulation variable: solvent reshapes DNA delivery

Yinghao Li[1, 2,†], Bei Qiu[1,3,†], Liang Yao[1,†], Jiahao Liu[6], Zishan Li[1,4], Xinhui Wang[5], Jing Lyu[1*], Wenxin Wang[1,6 *]

[1]Charles Institute of Dermatology, School of Medicine, University College Dublin, D04 V1W8 Dublin, Ireland.

[2]Yusuf Hamied Department of Chemistry, University of Cambridge, Cambridge, CB21EW, United Kingdom.

[3] Centre for Regenerative Medicine, Institute for Regeneration and Repair, the University of Edinburgh, Edinburgh, EH16 4UU, UK.

[4]Department of Chemistry, University of Manchester, Oxford Road, Manchester, M13 9PL UK, United Kingdom.

[5]School of Mechanical Engineering, State Key Laboratory of Traction Power, Southwest Jiaotong University, Chengdu 610031, China

[6]Institute of Precision Medicine (AUST-IPM), Anhui University of Science and Technology, Huainan 232001, China.

† These authors contributed to this work equally.

***Correspondence**: Jing Lyu, Wenxin Wang, Charles Institute of Dermatology, School of Medicine, University College Dublin, Dublin, D04 V1W8, Ireland.

Email: jing.lyu@ucd.ie; wenxin.wang@ucd.ie

1. Materials and Methods

1.1 Materials

For polymer synthesis and characterization, 1,4-Butanediol diacrylate (BDA), 5-amino-1-pentanol (AP), pentaerythritol tetraacrylate (PTTA), 1-(3-aminopropyl)-4-methylpiperazine (E7), 3-morpholinopropylamine, diaminopropane, 1,4-diaminobutane, hexamethylenediamine, 3-(Dimethylamino)-1-propylamine, 1,5-Diamino-2-methylpentane, 1,11-Diamino-3,6,9-trioxaundecane were purchased from Sigma-Aldrich. Lithium bromide (LiBr) for GPC measurements was purchased from Sigma-Aldrich. Dimethyl sulfoxide (DMSO), N-methyl-2-pyrrolidone (NMP), acetone, 1,3-butanediol, ethyl acetate, butanone, methyl isobutyl ketone, ethanol, and diethyl ether were purchased from Fisher Scientific. Deuterated chloroform ($CDCl_3$) and deuterated DMSO were purchased from Sigma-Aldrich. Hank's balanced salt solution, and alamarBlue Assay Kit purchased from Sigma and Invitrogen, respectively. Sodium acetate (Sigma) was diluted to 0.025 M prior to use. Cell culture Dulbecco's modified Eagle Medium (DMEM) was purchased from Sigma. Fetal bovine serum (FBS, Gibco, was filtered through 0.2 μm filters before use. gWiz-GFP commercial plasmid were obtained from Aldevron, Fargo, ND, USA.

1.2 Synthesis of PAEs

For the synthesis of the highly branched poly(β-amino ester) (HPAE) backbone, the monomers 1,4-butanediol diacrylate (BDA, 9.91 g), 5-amino-1-pentanol (AP, 5.15 g), and pentaerythritol tetraacrylate (PTTA, 1.76 g) were dissolved in dimethyl sulfoxide (DMSO) at a concentration of 30% (w/v). The reaction was carried out under argon protection. Once the polymer reached the desired molecular weight, the reaction

solution was evenly divided into eight portions, and different amines shown in **Figure 1** were added to each portion to terminate the reaction. The reaction mixtures were then diluted to 10% (w/v). Finally, the polymers were precipitated into diethyl ether for purification and dried under vacuum. All solvents and monomers were purchased from Thermo Fisher Scientific or Merck.

1.3 Polyplex Preparation

Generally, the polymers were initially dissolved in different solvent to stock solutions (100 mg/mL), and then the stock solutions were further diluted with 25 mM sodium acetate buffer according to the w/w ratio. As an example, for polymer/ DNA =20:1, the polymers were diluted to 2 mg/mL. DNA was diluted to 0.1 mg/mL with sodium acetate buffer. The polymer solutions were added into the DNA solution at equal volume, vortexed for 10 s, and allowed to stand for 15 min.

1.4 Size and Zeta Potential Assessment

The polyplex was prepared as mentioned above. After that, the sizes and zeta potentials of polyplexes were measured with a Malvern Panalytical Zetasizer (ZTS1240). All the measurements were repeated three times.

1.5 Cell Culture

CFBE41o- human CF bronchial epithelial and 16HBE14o- human bronchial epithelial cell lines were derived from a CF patient homozygous for the Phe508del Cystic Fibrosis Transmembrane Conductance Regulator (CFTR) mutation and a healthy donor, respectively, and both were immortalized with the origin-of-replication defective SV40 plasmid (pSVori-) (Merck). Cells were grown in flasks or plates coated with a

Fibronectin/Collagen/BSA extracellular matrix cocktail (Merck) in Minimum Essential Medium (MEM) cell culture medium (Merck) supplemented with 10% fetal bovine serum (FBS) (Thermo Fisher Scientific), 1X MEM Non-essential amino acid (only for CFBE41o- cell culture) (Merck), 2 mM glutamine (Thermo Fisher Scientific) and 100 U/ml penicillin, 100 µg/ml streptomycin sulphate (Thermo Fisher Scientific). All the cells were incubated at 5% CO_2 at 37°C.

1.6 Cell Transfection

Cells were seeded approximately 24 hours in advance at a density of around 20,000 cells per square centimeter. Transfections were conducted when the cell confluence reached 50%-70%. The transfection process involved introducing a series of HPAE polymers. The transfection took place in the culture medium, with plasmids at a final concentration of 5 ng/µL, previously complexed with the various synthesized polymers. The polymers and DNA were diluted in 25 mM Sodium Acetate (NaAc) at weight-to-weight (w/w) ratios of 10:1 and 20:1, then mixed at a 1:1 volume-to-volume (v/v) ratio. The expression of the reporter gene green fluorescent protein (GFP) was observed 48 hours post-transfection using an Olympus IX81 fluorescence microscope (Olympus, Tokyo, Japan). The intensity of GFP fluorescence was analyzed and semi-quantified using ImageJ software (NIH, Bethesda, MD, USA).

1.7 Cell Viability

AlamarBlue™ Kit (Thermo Fisher Scientific) was used to assess the cell viability. The method has been previously described. In brief, the cell viability was tested 48 h post-transfection by replacing the previous medium with fresh medium containing 10% (v/v)

alamarBlue™ reagent. A SpectraMax M3 multi-plate reader (Molecular Devices, San Jose, CA, USA) was used for Excitation/Emission values determination, which was recorded at 570 nm and 590 nm after incubating cells with 10% alamarBlue™ for 2 hours protected from light at 37 ℃. The untreated cells were used to normalize fluorescence values and set as 100% cell viability.

1.8 Western Blot Analysis

At 48 hours post-transfection, cells were rinsed with DPBS (Thermo Fisher Scientific), followed by the addition of radioimmunoprecipitation assay (RIPA) buffer (Thermo Fisher Scientific) containing protease inhibitor (Thermo Fisher Scientific) to lyse the cells and solubilize proteins. Protein quantification was conducted using the Pierce BCA Protein Assay Kit (Thermo Fisher Scientific). Subsequently, samples were loaded onto 7% NuPAGE Tris-Acetate protein gels (Thermo Fisher Scientific) and transferred to a nitrocellulose membrane (Thermo Fisher Scientific). For CFTR protein detection, the primary antibody, mouse anti-human CFTR UNC-596 (University of North Carolina, NC, USA), was diluted at 1:500, and the secondary antibody, anti-mouse IgG, HRP-linked (Cell Signaling, Danvers, MA, USA), was diluted at 1:2,000. Human α-tubulin (Cell Signaling) served as the loading control protein at a dilution of 1:1,000, with the secondary anti-rabbit IgG, HRP-linked antibody (Cell Signaling), diluted at 1:4,000. Protein bands were visualized and captured using Pierce™ ECL Western Blotting Substrate (Thermo Fisher Scientific) and the iBright CL750 Imaging System (Thermo Fisher Scientific). Semi-quantitative analysis of CFTR was performed using ImageJ Fiji software (NIH).

1.9 Animal studies

All animal procedures were conducted in accordance with protocols approved by the Biomedical Research Ethics Committee of Anhui University of Science and Technology (Approval No. SZ2024-004). Female BALB/c mice (8-10 weeks old, 18-22 g body weight) were obtained from Hangzhou Ziyuan Laboratory Animal Technology Co., Ltd. and housed under standard specific pathogen-free (SPF) conditions with ad libitum access to food and water. The nanoparticles, with a Polymer to DNA ratio of 20μg/1μg and a DNA concentration of 300μg/mL, were intravenously administered via tail vein injection at a volume of 200μL per mouse. Bioluminescence imaging was performed using the Tanon ABL-X5 in vivo imaging system, with mice receiving an intraperitoneal injection of 150 μL D-luciferin (30 mg/mL in PBS) at 6 and 24 hours post-nanoparticle injection. Signal intensities were quantified using Tanon Image Analysis Software (version Tanon Prime-5000 In Vivo Imaging System).

2. Supplementary Figures

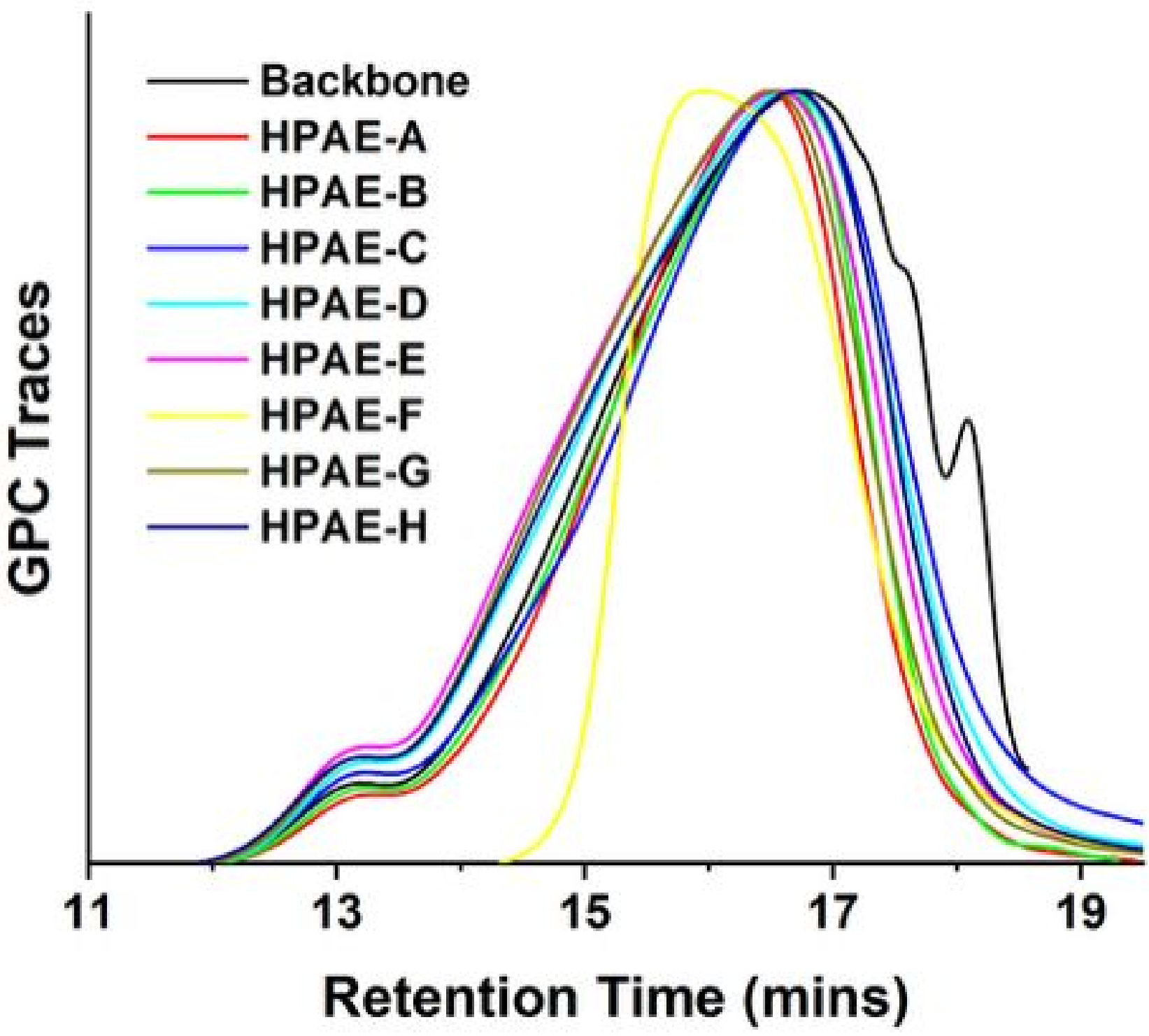

Figure S1. GPC traces of HPAE-A to -H, and HPAE backbone before end-capped.

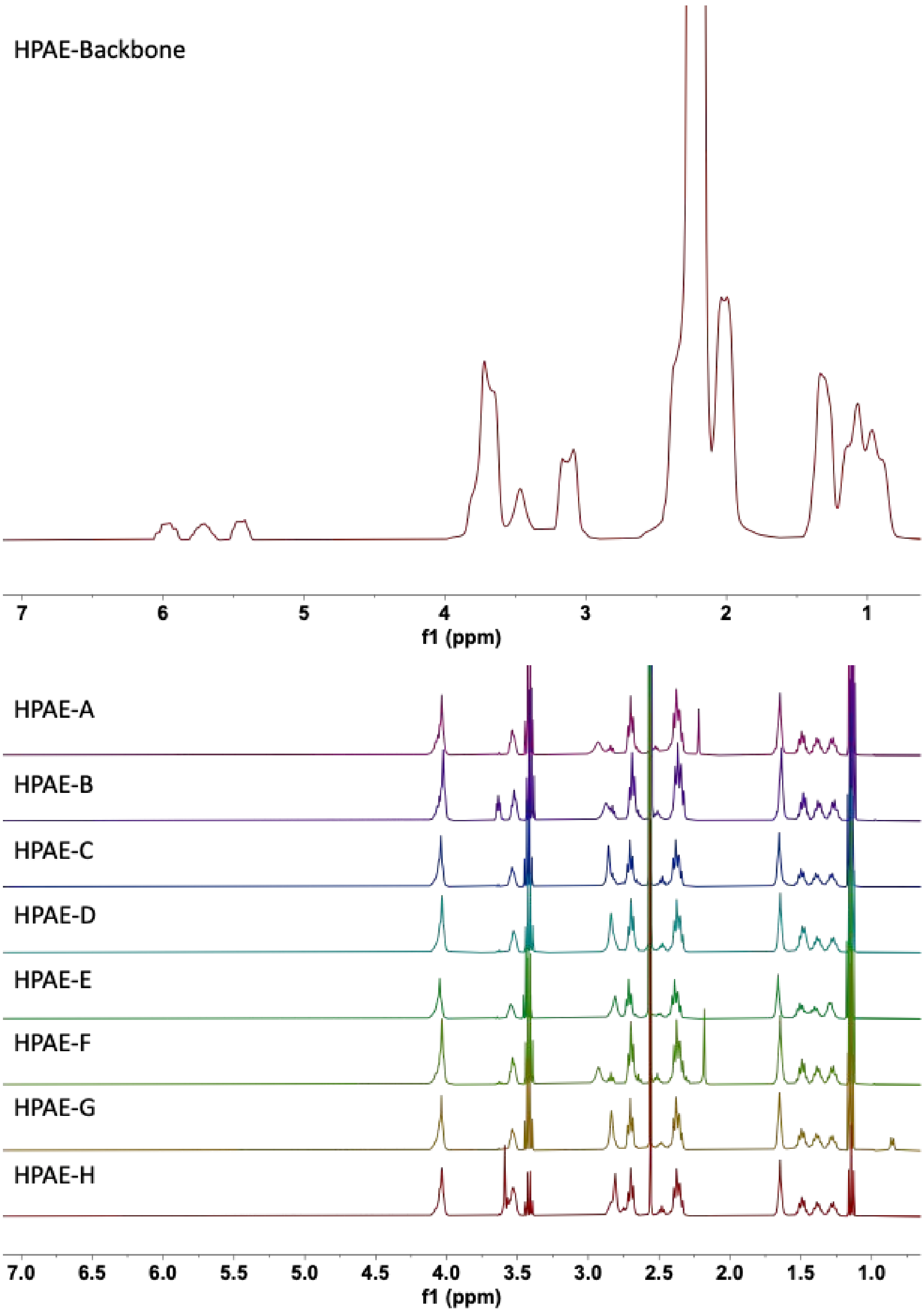

Figure S2. ^{1}H NMR spectra of HPAE-A to -H, and HPAE backbone before end-capped.

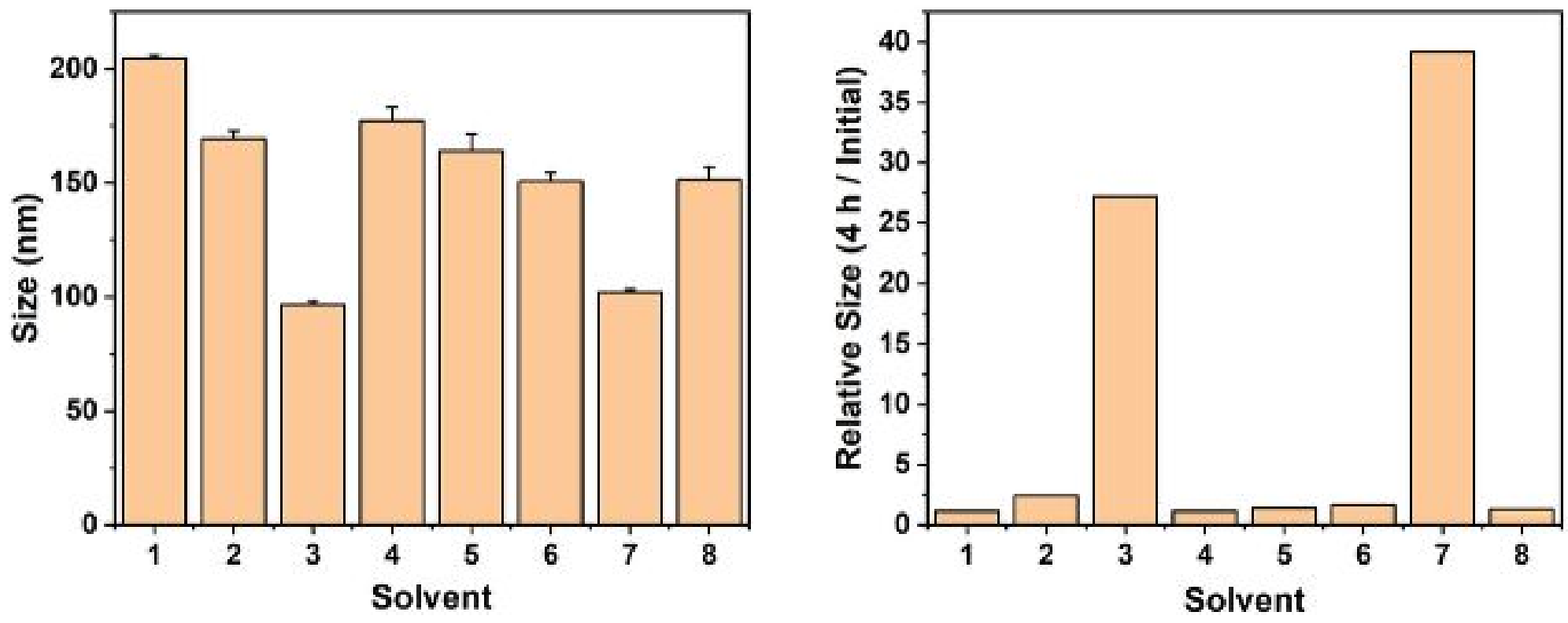

Figure S3. Size and stability of HPAE-A polyplexes in different solvent systems. (Left) Size of HPAE-A polyplexes prepared at a polymer:DNA weight ratio (w/w) of 20:1. (Right) Relative size change after 4 h incubation at room temperature (normalized to initial size).

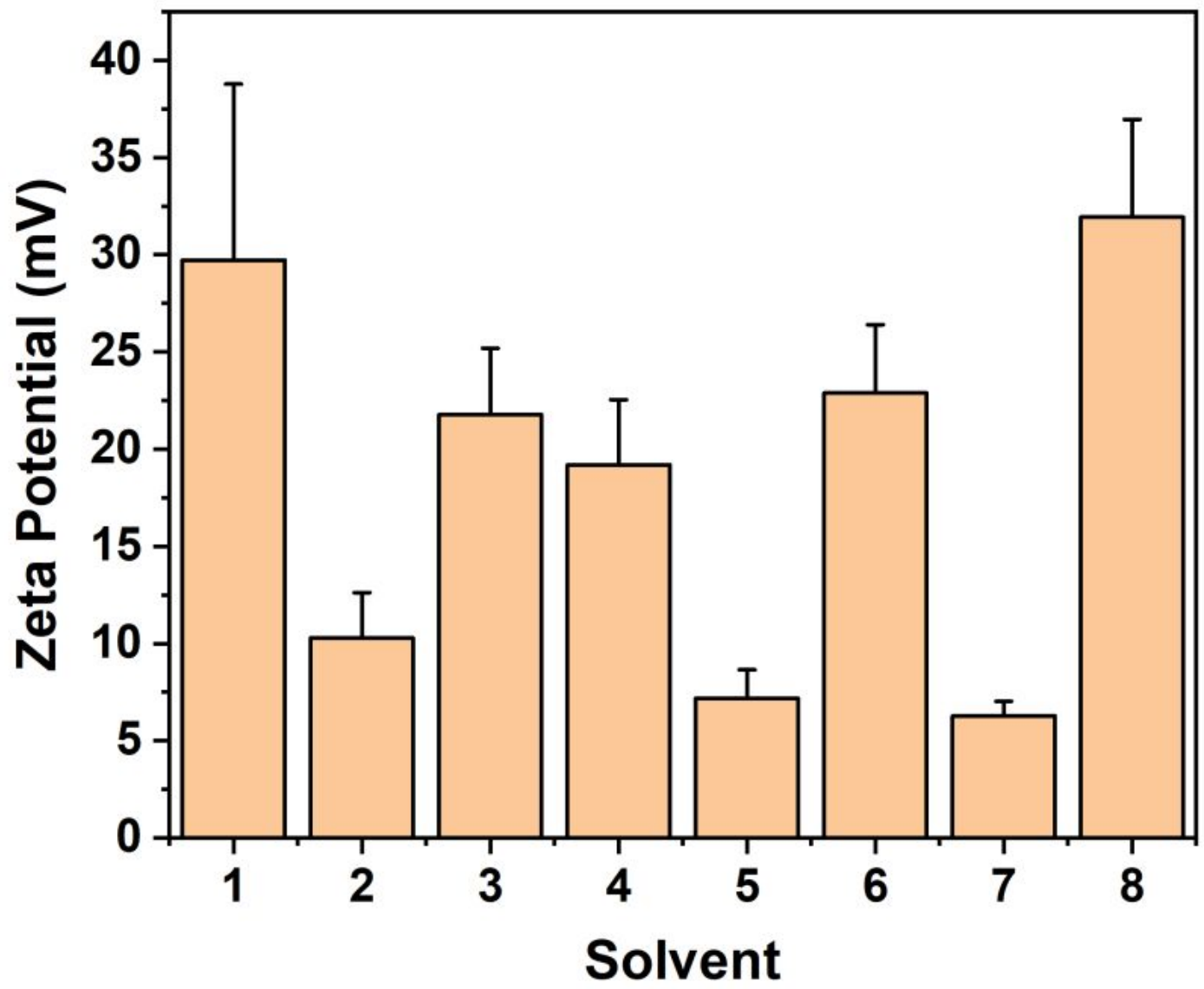

Figure S4. Zeta potential of HPAE-A polyplexes in different solvent systems.

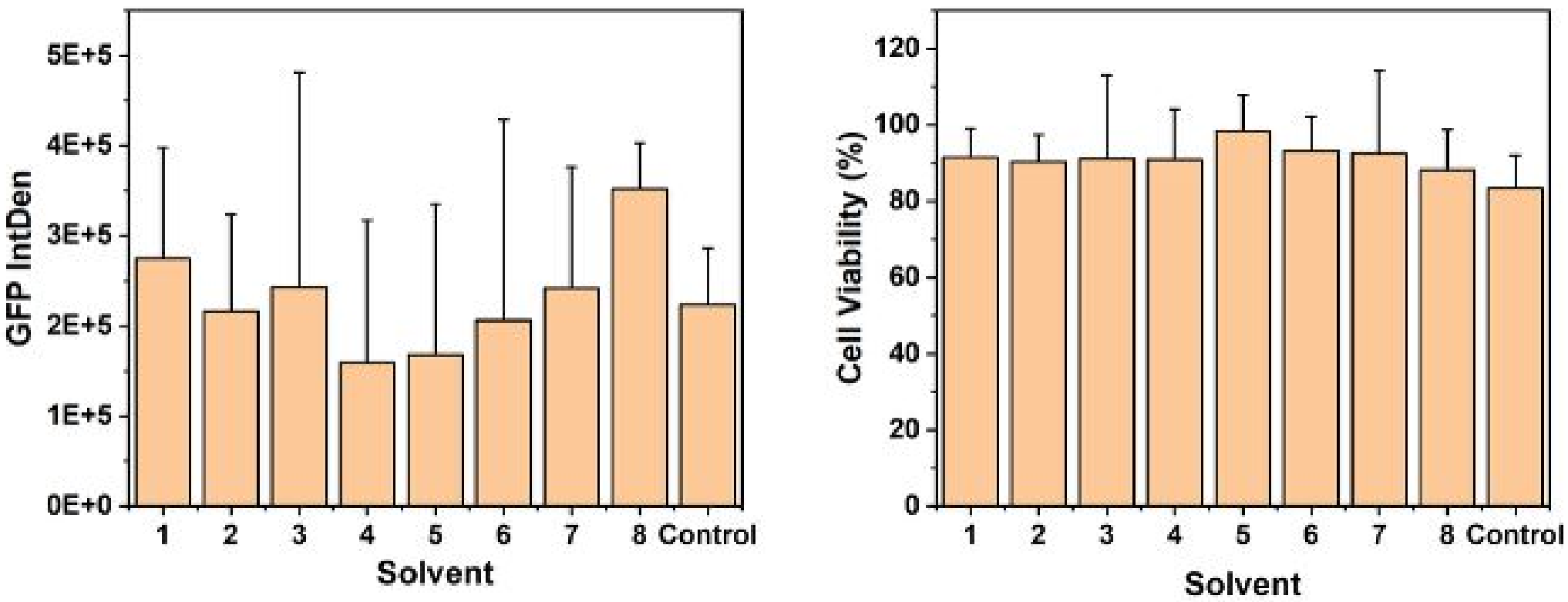

Figure S5. *In vitro* transfection performance and cell viability of HPAE-A in different solvent systems. (Left) Transfection efficiency at a polymer:DNA weight ratio (w/w) of 20:1. (Right) Cell viability after 48 h post-transfection. The control group was A1 (HPAE-A in solvent 1, DMSO) without additional solvent.

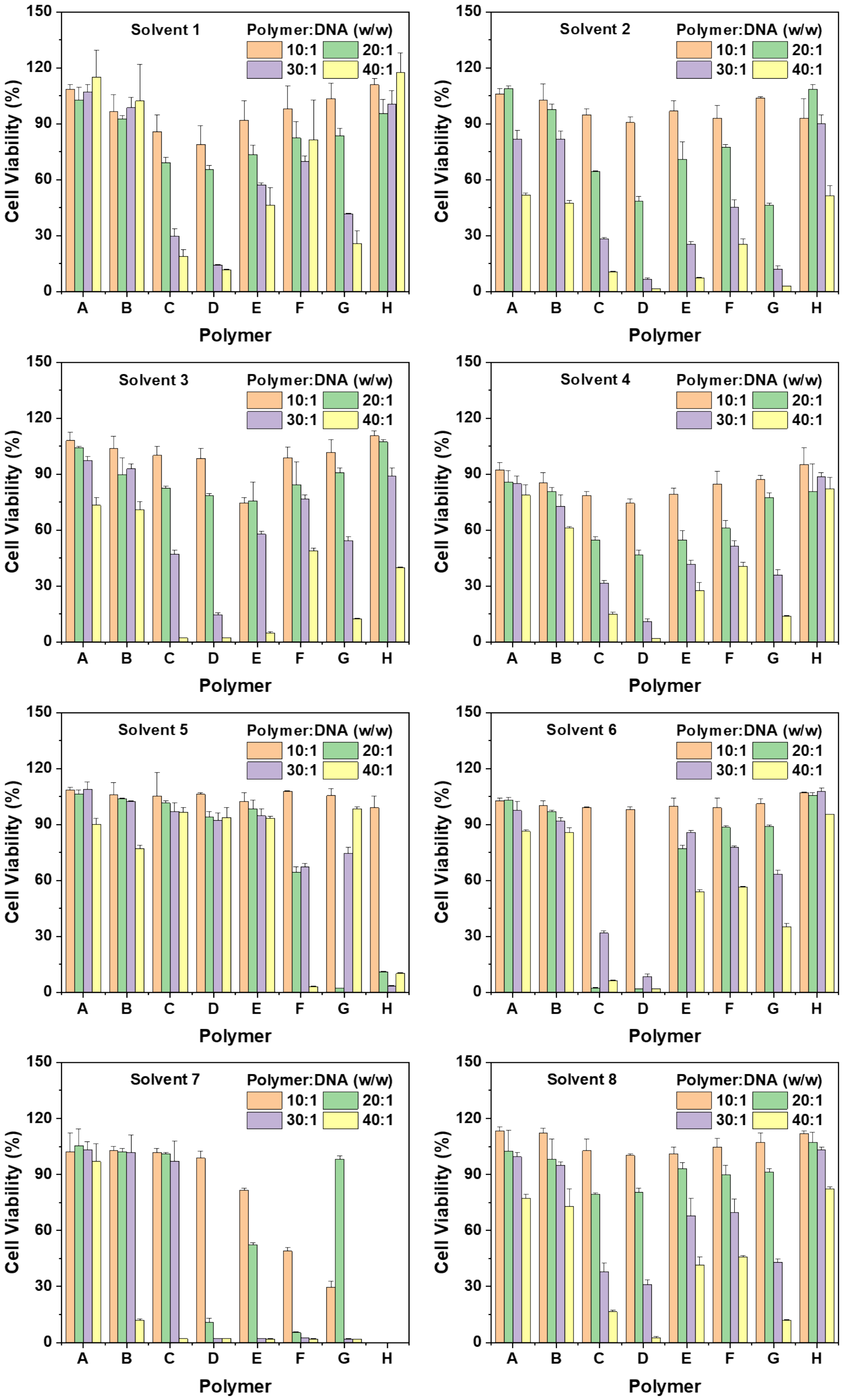

Figure S6. Cell viability of polymers stored in different stock solvents after 48 h of transfection.

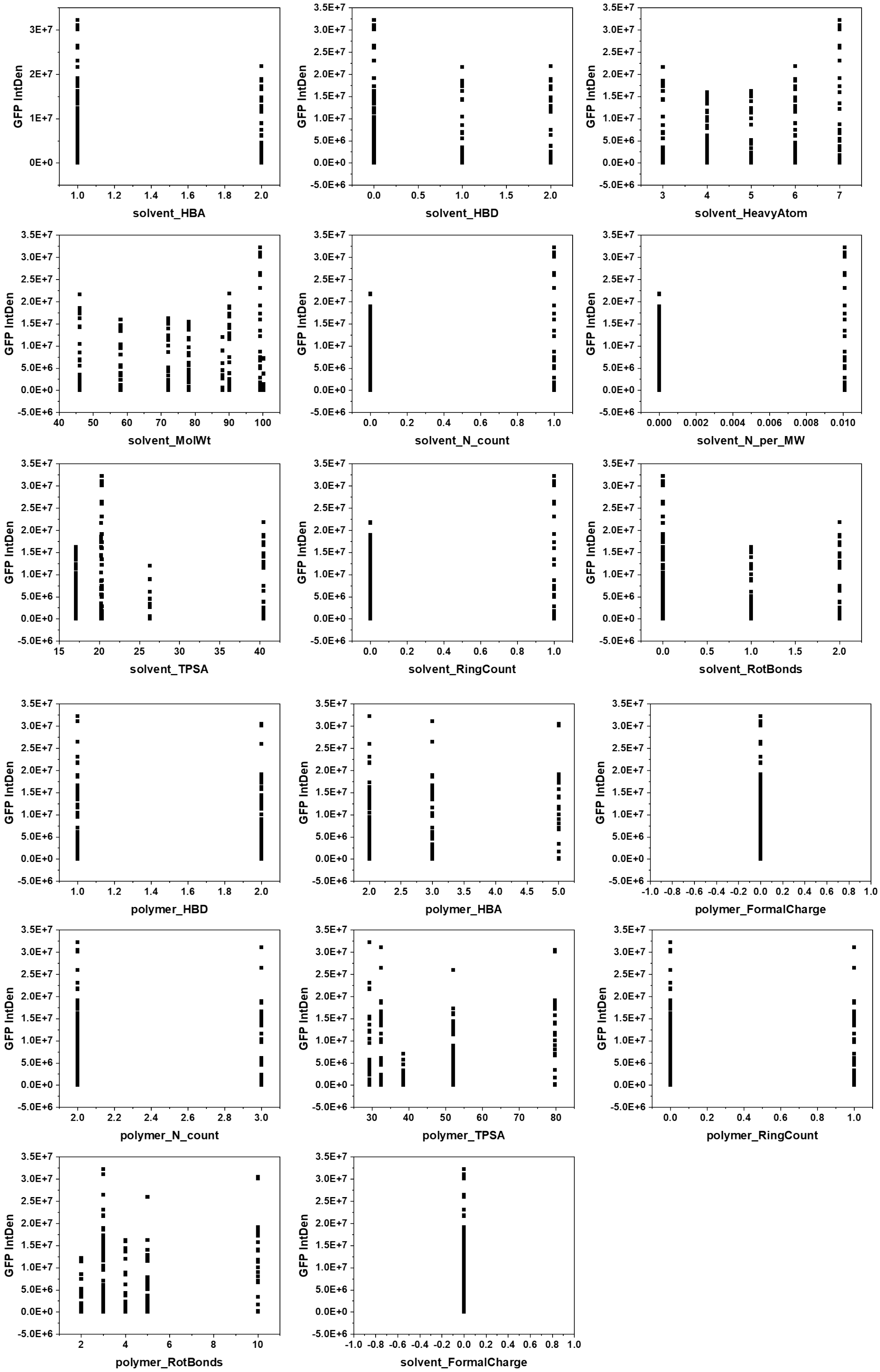

Figure S7. Relationships between polymer/solvent features and transfection efficiency.

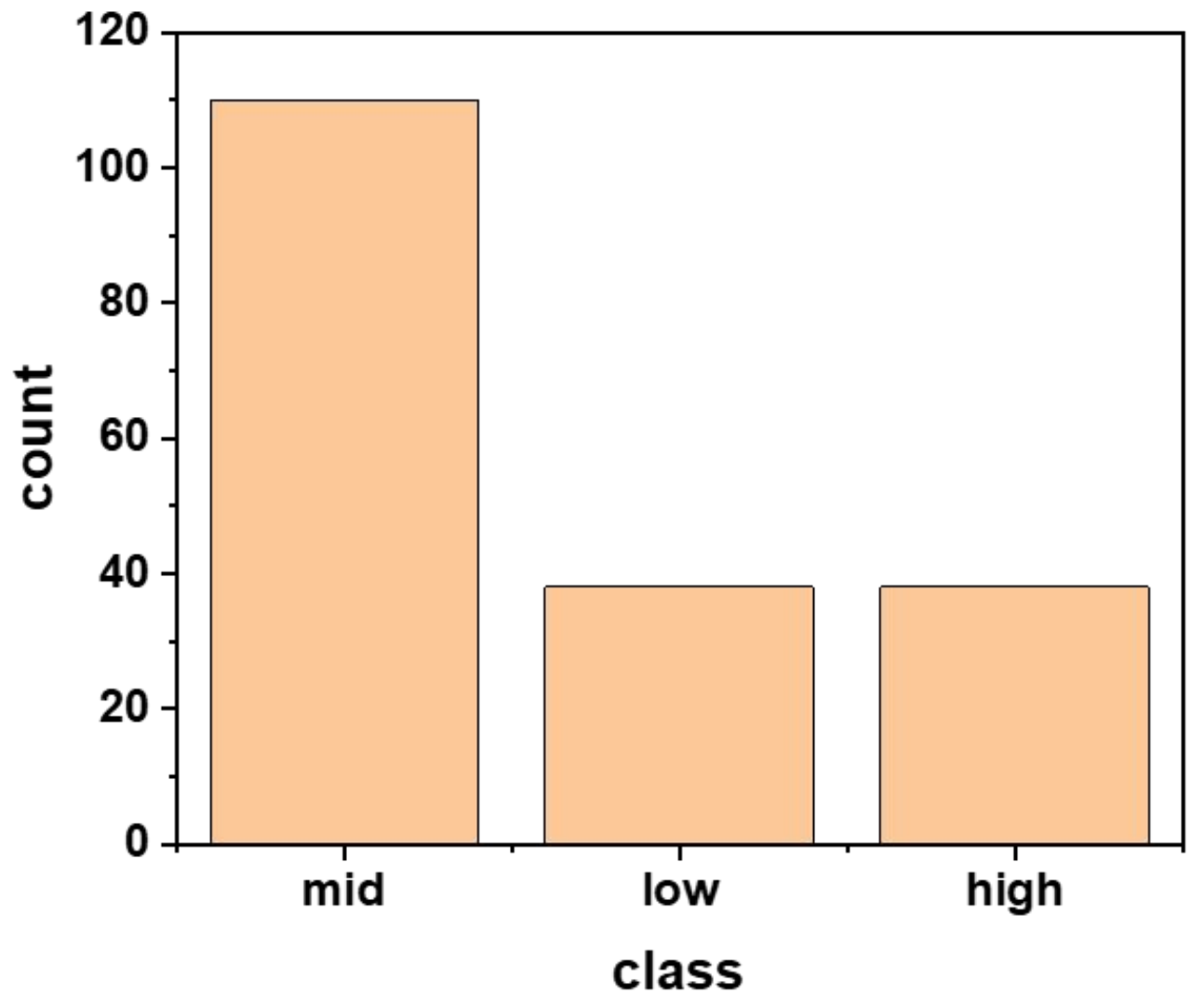

Figure S8. Quantile-based classification of high, mid, and low transfection performers.

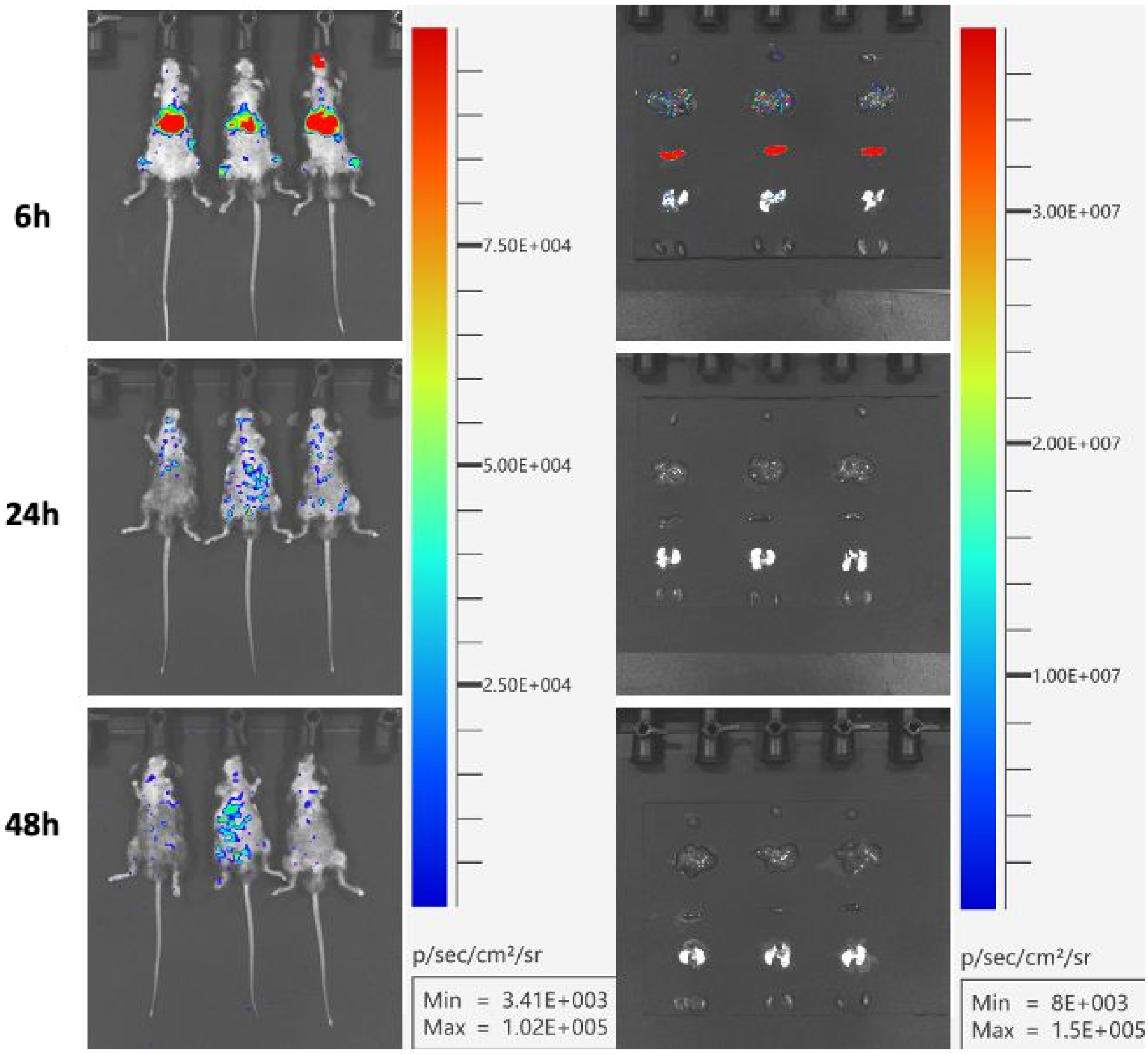

Figure S9. *In vivo* transfection performance evaluation with HPAE-A in solvent 1 (DMSO). (Left) Bioluminescence images of immunocompetent mice and luminescence images of major organs at different time points after tail-vein injection of polymer complexes. (Right) Organs images, with organs arranged from top to bottom as heart, liver, spleen, lung, and kidney.

Table S1. GPC characterization results.

	M_p (g/mol)	M_n (g/mol)	M_w (g/mol)	$Đ$
**HPAE-Backbone**	3017	2806	11211	4.0
**HPAE-A**	3884	3996	12275	3.1
**HPAE-B**	3369	3856	12580	3.2
**HPAE-C**	3163	2611	11253	4.3
**HPAE-D**	3317	3125	13167	4.2
**HPAE-E**	3589	3553	14808	4.1
**HPAE-F**	6339	2960	5633	1.9
**HPAE-G**	3763	3790	14642	3.8
**HPAE-H**	3213	3334	14125	4.2
